# Age-related differences in motives for contacting out-of-hours primary care: a cross-sectional questionnaire study in Denmark

**DOI:** 10.1080/02813432.2020.1794160

**Published:** 2020-07-23

**Authors:** Grete Moth, Morten B. Christensen, Helle Collatz Christensen, Anders H. Carlsen, Ingunn S. Riddervold, Linda Huibers

**Affiliations:** a Research Unit for General Practice, Aarhus, Denmark; b Emergency Medical Services Copenhagen, Ballerup, Denmark; c Prehospital Emergency Medical Services, Aarhus, Denmark

**Keywords:** Out-of-hours care, primary care, help–seeking behavior, motivation, age factors

## Abstract

**Objective:**

Demands for out-of-hours primary care (OOH-PC) services are increasing. Many citizens call because of non-urgent health problems. Nevertheless, the patients’ motives for requesting medical help outside office hours remains an understudied area. This study aimed to examine motives for calling OOH-PC services in various age groups.

**Design:**

Cross-sectional paper based questionnaire study conducted during two weeks in 2015.

**Setting:**

The OOH-PC services in two Danish regions.

**Subjects:**

Randomly selected patients calling the two healthcare services and accepting to participate in the study received a questionnaire on patient characteristics, health problems, and 26 pre-defined motives based on the Andersen Behavioural Model. Multivariate regression analyses were conducted for various age groups to calculate the probability of each motive to be a significant factor for the decision to call.

**Results:**

A total of 1,871 patients were included in the study; half were parents of children aged 0-12 years. Young adults (18 to 39 years) differed significantly from other age groups as they more often stated perceived barriers and benefits such as “Own GP no time available soon enough” and “Need for quick help because of work”.

**Conclusion:**

Young adults more often perceive barriers and benefits, which may suggest af difference in expectations regarding the purpose of out-of-hours services and accessibility. Further research is needed to address this issue and further explore the potential gap between the citizens’ expectations to the OOH-PC services and the prevailing health policies.

**Key points**
- The out-of-hours primary healthcare services are increasingly contacted for non-urgent problems, but little is known about the citizens’ motives for calling.
- Age is associated with differences in the perceived importance of various motives for calling out-of-hours care.
- Young adults are more often than other age groups motivated to call due to logistical issues, such as their job.

## Introduction

Most western countries offer out-of-hours primary care (OOH-PC) services for their citizens. These services are intended to target urgent medical needs that cannot wait until the next day [1]. Recent years have seen a general increase in the demands for healthcare services, including OOH-PC services [2,3], and a large proportion of these contacts concern non-urgent health problems [4,5]. This development has caused excessive demands on OOH-PC services, which may result in increased use of resources, longer waiting times, and risk of treatment delay for acute health problems [2,6].

The use of OOH healthcare has shown to be higher for young children than for adults; this is seen across countries and healthcare organisations [5,7,8]. Moreover, the reasons for encounter differ for different age groups [5,9,10]. Parents often call for medical help and advice concerning their children due to infection-related symptoms, whereas older patients have larger variation of reasons for encounter [11]. Likewise, various motives are likely to play different roles for different age groups. However, knowledge is sparse on the motives for requesting out-of-hours care across age groups. Thus, gaining more knowledge on the patients’ motives for calling is crucial to improve the healthcare services and meet the increasing demands for OOH-PC.

In Denmark, the healthcare personnel managing the OOH-PC have no prior knowledge of the contacting patients contrary to daytime primary care, where the general practitioner (GP) has profound knowledge of each patient’s previous medical record and current personal challenges [12]. The patient plays an important role for the increasing demands on the OOH-PC services as the decision to call for medical help lies with the patient. Apart from the acute health problem itself, other motives may play a role for the patient’s decision to contact the OOH-PC services, such as perceived lack of control or excessive worry [13,14]. Moreover, limited access to the daytime GP has been associated with higher use of OOH-PC services [15].

The present study aimed to examine the importance of different patient motives for seeking medical advice outside office hours at the OOH-PC services for various age groups.

## Methods

### Design and setting

A cross-sectional study was conducted to explore the patients’ motives for contacting the OOH-PC services. As patients in Denmark have direct access to both the OOH-PC services and the prehospital EMS for acute health problems outside office hours, our study population consisted of patients contacting one of these in two regions. This article will focus on the patients contacting an OOH-PC service: the GP cooperative (GPC) in the Central Denmark Region and the medical helpline 1813 (MH-1813) in the Capital Region of Denmark, Copenhagen during two weeks in February and March 2015.

Five administrative regions in Denmark are responsible for organising OOH-PC on weekdays from 4 pm to 8am, weekends, and holidays. In each region, OOH healthcare is provided by primary healthcare, emergency departments (EDs), and the prehospital emergency medical services (EMS) [12,16]. The OOH services in the two included regions use different organisation models. The Central Denmark Region has a, large-scale GP cooperative (GPC). GPs answer calls directly and perform the triage of patients, either giving telephone advice or referring the patient to a subsequent face-to-face consultation [1]. In the Capital Region of Denmark, OOH-PC as an integrated part of the EMS is the entrance for non-urgent cases in the form of a medical helpline 1813 (MH-1813) staffed by nurses to perform the triage. The nurses use a computerised decision-support tool and also have the opportunity to consult a doctor. Patients receive telephone advice or are referred to a face-to-face consultation.

OOH-PC is tax-funded in Denmark and thus free of charge for the patients.

### Population

Patients contacting the OOH-PC services in the study period were met by a message on the telephone waiting line informing them about the ongoing project and they could decline participation by pressing ‘9’. A random selection of patients accepting to participate were included. Only the first contact of each patient was included to ensure that each patient was only represented once in the study population. Patients with anonymized address, living in an institution, and aged 13–18 years were excluded as were tourists and citizens with an invalid civil registration number.

### Questionnaire

A systematic literature search was conducted to identify studies on factors related to decision-making in patients and their motives for contacting OOH-PC. A questionnaire on patient motives for contacting healthcare was developed, inspired by the Andersen Behavioural Model [17] and adapted to the Danish healthcare system. The questionnaire contained questions on the main characteristics of the decision maker (age, gender, ethnicity, marital status, education, and self-perceived overall health) and the health problem. Motives were measured with 26 predefined statements on the decision to contact the OOH-PC services; the importance of each statement for the decision to call was rated on a 5-point Likert scale (see the questionnaire in Supplement 1).

Based on our model, the motives were categorised into five main groups: ‘own assessment and expectations’, ‘perceived barriers and benefits’, ‘previous experience and knowledge’, and ‘needs and wishes’ (see Supplement 1). The questionnaire was tested and modified based on interviews with patients. The final version was validated in three small-scale pilot studies. This procedure has been thoroughly described elsewhere [18].

### Data collection

A power calculation was performed to ensure identification of a 10% difference between the two settings in the importance of motives, which was the overall aim of the study For this we needed 400 respondents per setting for children as well as for adults. As we in the final pilot test obtained a response rate of 40% we aimed to send out 1000 questionnaires per setting for children as well as for adults. Data on calls to the OOH-PC service were received electronically twice a week. Questionnaires were sent to patients within four days after their OOH contact to minimise the risk of recall bias, including details of the situation and considerations prompting the call. The enclosed invitation letter included link and login to a web-based version of the questionnaire. A reminder was sent to non-respondents by mail after two weeks. Questionnaires for patients below age 13 years were sent to the parents, who were asked to fill out the questionnaire also in cases in which others were involved in calling the OOH-PC service.

### Data management

Motives were dichotomised into ‘not important’ (‘not relevant’, ‘no importance’, ‘little importance’, ‘some importance’) and ‘important’ (‘important’, ‘very important’). Moreover, before inclusion in the analyses, ethnicity was categorised into ‘native Danes’,’ western immigrants’, and ‘other immigrants’ based on answers regarding where the patient and parents were born. Educational level was categorised into ‘low’, ‘medium’, and ‘high’ based on the highest level of completed education. Marital status was dichotomised into ‘in a relationship’ (‘married’, ‘cohabiting’ or ‘committed relationship’) or ‘single’. Self-assessed health was dichotomised into ‘good’ (‘excellent’,’ very good’, ‘good’) and ‘poor’ (‘less good’, ‘bad’).

### Statistical analyses

First, descriptive analyses of the main characteristics of the respondents and all the motives were performed and stratified by age group. Next, we conducted multivariate regression analyses using a generalized linear model (GLM) to obtain prevalence rate ratios (PRR) of the probability for each motive to be a significant motive for the decision to call. The age group 18–39 years was used as a reference category. The latter analyses were adjusted for ethnicity, education level, marital status, and self-assessed health status with a significance level of 95%. Stata 14 (StataCorp LP, College Station, TX, USA) was used for the statistical analyses.

## Results

### Descriptive characteristics

A total of 1871 patients contacting OOH-PC services were included in the study (response rate: 47.1%). A non-response analysis showed that respondents did not differ from non-respondents on gender. Parents of young children and young adults aged 18–39 years chose statistically significantly more often than other groups not to respond (53 and 64%, respectively), whereas 62% of the patients aged >64 years responded (data not shown). Differences in response rates were seen between the two regions. The response rates for the age groups 18–39 years and 40–64 years were statistically significantly lower in the Capital Region of Denmark compared with the Central Denmark Region (data not shown).

Half of the respondents were parents of children aged 0–12 years (Table 1). Across age groups, we found that parents of the included children were significantly more often in a relationship (0–4 years: 91.7% and 5–12 years: 85.4%) compared with adult patients (range: 53.0–72.6%). Parents of included children also significantly more often had a high education (61.9 and 56.3%) compared with adult patients (range: 21.4–44.8%). Concerning ethnicity, we also found statistically significant differences between age groups as the share among parents to 5–12 year old children was 80.9% compared with the share of native Danes among adults (84.1–85.3%).

**Table 1.** Baseline information on age, ethnicity, marital status, education, and self-perceived health for the included patients.

Age group	0–4 years (parents)	5–12 years (parents)	18–39 years	40–64 years	>64 years	All	Test of difference *p*-value
	*N* (%)	*N* (%)	*N* (%)	*N* (%)	*N* (%)		
	604 (32.3)	357 (19.1)	327 (17.5)	368 (19.7)	215 (11.5)	1875 (100)	
Share of male patients	314 (52.0)	177 (49.6)	106 (32.4)	143 (38.9)	94 (43.7)	834 (44.6)	<0.001
Marital status							
Single	45 (7.5)	43 (12.0)	92 (28.1)	88 (23.9)	88 (40.9)	356 (19.0)	<0.001
In a relationship	554 (91.7)	305 (85.4)	226 (69.1)	267 (72.6)	114 (53.0)	1466 (78.4)	
Missing inf.	5 (0.8)	9 (2.5)	9 (2.8)	13 (3.5)	13 (6.1)	49 (2.6)	
Ethnicity							
Native Danes	512 (84.8)	289 (80.9)	275 (84.1)	313 (85.3)	182 (84.6)	1571 (84.0)	<0.001
Western immigrants	40 (6.6)	31 (8.7)	20 (6.1)	22 (6.0)	10 (4.7)	123 (6.6)	
Other immigrants	40 (6.6)	26 (7.3)	25 (7.7)	18 (4.9)	1 (0.5)	110 (5.9)	
Missing inf.	12 (2.0)	11 (3.1)	7 (2.1)	15 (4.1)	22 (10.2)	67 (3.6)	
Education							
Low	30 (5.0)	16 (4.5)	45 (13.8)	37 (10.1)	49 (22.8)	177 (9.5)	<0.001
Middle	193 (32.0)	130 (36.4)	162 (49.5)	152 (41.3)	102 (47.4)	739 (39.5)	
High	374 (61.9)	201 (56.3)	114 (34.9)	165 (44.8)	46 (21.4)	900 (48.1)	
Missing inf.	7 (1.2)	10 (2.8)	6 (1.8)	14 (3.8)	18 (8.4)	55 (2.9)	
Self-perceived health							
Poor	29 (4.8)	21 (5.9)	50 (15.3)	74 (20.1)	81 (37.7)	255 (13.6)	<0.001
Good	567 (93.9)	325 (91.0)	269 (82.3)	285 (77.5)	118 (54.9)	1,563 (83.6)	
Missing inf.	8 (1.3)	11 (3.1)	8 (2.4)	9 (2.5)	16 (7.4)	52 (2.8)	

### Importance of motives

The importance of different motives to each age group is presented in Table 2. For all motives, we found marked differences between the age groups. However, age group 18–39 years stood out for several of the motives related to perceived barriers and benefits. Young adults reported to find it more difficult to get access to their own GP on the telephone (16.2%) compared with other age groups (range: 8.2–12.7%) and to book a consultation (18–39 years: 22.4%; other age groups: range 13.5–14.9%). They also more often considered calling OOH as the easiest option (19.0% compared with range 10.1–13.6%). Furthermore, a larger part of the young adults stated ‘recommended to call’ as an important motive (40.7%) compared with other age groups (range: 9.5–27.0%).

**Table 2.** The importance of motives in different age groups.

Age groups*	0–4 years (*N* = 604)		5–12 years (*N* = 357)		18–39 years (*N* = 327)		40–64 years (*N* = 368)		>64 years (*N* = 215)	
	*N* (%)	*N* (%)	*N* (%)	*N* (%)	*N* (%)	*N* (%)	*N* (%)	*N* (%)	*N* (%)	*N* (%)
	Not important	Important	Not important	Important	Not important	Important	Not important	Important	Not important	Important
Own assessments and expectations										
Perceived need for prompt action	117 (19.4)	477 (79.0)	71 (19.9)	283 (79.3)	73 (22.3)	251 (76.8)	46 (12.5)	307 (83.4)	28 (13.0)	156 (72.6)
Unpleasant symptoms	122 (20.2)	474 (78.5)	68 (19.1)	284 (79.6)	53 (16.2)	271 (82.9)	53 (14.4)	301 (81.8)	24 (11.2)	173 (80.5)
Perceived condition to be life-threatening	553 (91.6)	39 (6.5)	327 (91.6)	21 (5.9)	298 (91.1)	22 (6.7)	296 (80.4)	41 (11.1)	110 (51.2)	37 (17.2)
Worried	214 (53.4)	381 (63.1)	173 (48.5)	180 (50.4)	141 (43.1)	183 (56.0)	143 (38.9)	206 (56.0)	53 (24.7)	128 (59.5)
Expected need for examination	410 (67.9)	183 (30.3)	196 (54.9)	154 (43.1)	214 (65.4)	108 (33.0)	192 (52.2)	145 (39.4)	79 (36.7)	71 (33.0)
Expected need for specialist care/hospital admission	519 (85.9)	74 (12.3)	298 (83.5)	50 (14.0)	274 (73.8)	50 (15.3)	255 (69.3)	82 (22.3)	85 (39.5)	66 (30.7)
Expected need for ambulance	587 (97.2)	4 (0.7)	344 (96.4)	3 (0.8)	313 (95.7)	7 (2.1)	319 (86.7)	18 (4.9)	116 (54.0)	60 (3.2)
Perceived barriers and benefits										
Own GP not accessible during daytime on the telephone	517 (85.6)	76 (12.7)	309 (86.6)	41 (11.5)	265 (81.0)	53 (16.2)	310 (84.2)	30 (8.2)	131 (60.9)	225 (12.0)
Own GP no time available soon enough	501 (83.0)	89 (14.7)	298 (83.5)	53 (14.9)	250 (76.5)	70 (22.4)	288 (78.3)	52 (14.1)	127 (59.1)	293 (13.5)
No possibility for the patient to contact own GP during daytime	538 (89.1)	52 (8.6)	325 (91.0)	26 (7.3)	280 (85.6)	39 (11.9)	313 (85.1)	25 (6.8)	137 (63.7)	16 (7.4)
Easiest option	510 (84.4)	82 (13.6)	311 (87.1)	36 (10.1)	259 (79.2)	62 (19.0)	288 (78.3)	48 (13.0)	103 (47.9)	48 (13.0)
Need arose outside office hours	49 (8.11)	551 (91.2)	29 (8.1)	326 (91.3)	43 (13.2)	280 (85.6)	33 (9.0)	329 (89.4)	32 (14.9)	157 (73.0)
Need for quick help because of work	520 (86.1)	71 (11.8)	319 (89.4)	32 (9.0)	209 (63.9)	109 (33.3)	253 (68.8)	88 (23.9)	144 (67.0)	13 (6.1)
Need for quick help because of daycare attendance	528 (87.4)	66 (10.9)	319 (89.4)	31 (8.7)	0 (0.0)	0 (0.0)	0 (0.0)	0 (0.0)	0 (0.0)	0 (0.0)
Previous experience and knowledge										
Previous experience with similar symptoms	418 (69.2)	178 (29.5)	218 (61.1)	134 (37.5)	230 (70.3)	91 (27.8)	226 (61.4)	125 (34.0)	83 (38.6)	84 (39.1)
Previous positive experience with this healthcare service	352 (58.3)	244 (40.4)	228 (63.9)	125 (35.0)	218 (66.7)	101 (30.9)	235 (63.9)	115 (31.3)	98 (45.6)	65 (30.2)
Perceived most suitable healthcare service	146 (24.2)	452 (74.8)	89 (24.9)	265 (74.2)	104 (31.8)	219 (67.0)	71 (19.3)	291 (79.1)	29 (13.5)	158 (73.5)
Needed second opinion	563 (93.2)	30 (5.0)	339 (95.0)	13 (3.6)	305 (93.3)	15 (4.6)	333 (90.5)	11 (3.0)	145 (67.4)	12 (5.5)
Needs and wishes										
Did not know what to do	329 (54.5)	265 (43.9)	220 (61.6)	131 (36.7)	145 (44.3)	174 (53.2)	194 (52.7)	149 (40.5)	74 (34.4)	85 (39.5)
Did not know where else to call	416 (68.9)	179 (29.6)	267 (74.8)	80 (22.4)	192 (58.7)	127 (38.8)	236 (64.1)	104 (28.3)	81 (37.7)	75 (34.9)
Wanted to talk to a physician	150 (24.8)	449 (74.3)	95 (26.6)	258 (72.3)	93 (28.4)	228 (69.7)	82 (22.3)	270 (73.4)	27 (12.6)	149 (69.3)
Could not take responsibility	442 (73.2)	152 (25.2)	246 (68.9)	103 (28.9)	207 (63.3)	113 (34.6)	200 (54.4)	145 (39.4)	60 (27.9)	103 (32.9)
Nobody to talk to	575 (95.2)	20 (3.3)	332 (93.0)	18 (5.0)	298 (91.1)	20 (6.1)	320 (87.0)	19 (5.2)	128 (59.3)	24 (11.2)
Recommended (from non-medical person) to call	529 (87.6)	68 (11.3)	317 (88.8)	34 (9.5)	183 (56.0)	133 (40.7)	281 (76.4)	64 (17.4)	98 (45.6)	58 (27.0)

*The number of ‘Not relevant’ responses and missing information vary for each variable and are not shown, as they account for only few of the answers.

Factors related to own assessments and expectations, such as ‘expected need for specialist care/hospital admission’ was more often an important motive in the older age groups (40–60 years: 22.3% and >64 years: 30.7%) compared with the younger age groups (range: 12.3–15.3%).

For children, ‘perceived need for prompt action’, ‘unpleasant symptoms’, ‘worried’, ‘wanted to talk to a physician’ as well as ‘perceived most suitable health care service’ were in many cases important motives for young children (range from 63.1 to 79.0%) as well as for children aged 5–12 years (range from 50.4 to 79.3%).

### Motives related to the decision to call

The results of the regression analysis of the adjusted association between age and various motives are presented in Table 3. Young adults (18–39 years, reference category) differed from other age groups for several motives, in particular motives related to perceived barriers and benefits. A predominant motive for young adults was ‘Own GP no time available soon enough’ compared with most other age groups (PRR: 0–4 years: 0.73 (95% confidence interval (CI): 0.54–0.98), 5–12 years: 0.71 (CI: 0.51–1.00), 40–64 years: 0.64 (CI: 0.48–0.94)). ‘Need for quick help because of work’ was also a strong motive for young adults compared with all other age groups (PRR: 0–4 years: 0.25 (CI: 0.17–0.36), 5–12 years: 0.17 (CI: 0.10–0.26), 40–64 years: 0.66 (CI: 0.46–0.93), >64 years: 0.15 (CI: 0.07–0.30)). As differences were seen in the response rates between the two regions for the group of young adults as mentioned above, we performed additional analyses to examine if the responses to these two questions differed for the two regions. This was not the case (data not shown).

**Table 3.** Prevalence rate ratios of the adjusted association between age groups and the importance of the various motives.

	Adjusted prevalence rate ratio* (95% confidence interval)				
	0–4 years	5–12 years	18–39 years (reference group)	40–64 years	>64 years
Own assessments and expectations					
Perceived need for prompt action	1.04 (0.96–1.12)	1.03 (0.95–1.12)	1	1.14 (1.06–1.22)	1.09 (0.99–1.20)
Unpleasant symptoms	0.82 (0.56–1.20)	0.81 (0.54–1.23)	1	1.11 (0.73–1.71)	1.43 (0.81–2.53)
Perceived condition to be life-threatening	0.99 (0.59–1.68)	0.84 (0.45–1.54)	1	1.75 (1.06–2.88)	3.51 (2.07–5.94)
Worried	1.54 (1.15–2.06)	0.90 (0.66–1.24)	1	1.23 (0.89–1.68)	1.67 (1.10–2.53)
Expected need for examination	0.90 (0.74–1.11)	1.28 (1.04–1.56)	1	1.32 (1.08–1.61)	1.52 (1.19–1.93)
Expected need for specialist care/hospital admission	0.84 (0.55–1.27)	0.88 (0.56–1.39)	1	1.87 (1.24–2.83)	4.16 (2.57–6.74)
Expected need for ambulance	0.45 (0.13–1.61)	0.36 (0.07–1.77)	1	3.05 (1.22–7.62)	8.60 (3.38–1.88)
Renewal of prescription	0.59 (0.33–1.06)	0.22 (0.09–0.59)	1	0.94 (0.53–1.64)	0.67 (0.29–1.54)
Perceived barriers and benefits					
Own GP not accessible during daytime on the telephone	0.89 (0.63–1.25)	0.73 (0.49–1.09)	1	0.53 (0.34–0.82)	1.03 (0.66–1.61)
Own GP no time available soon enough	0.73 (0.54–0.98)	0.71 (0.51–1.00)	1	0.67 (0.48–0.94)	0.85 (0.57–1.27)
No possibility for the patient to contact own GP during daytime	0.76 (0.50–1.14)	0.60 (0.37–0.98)	1	0.60 (0.37–0.98)	0.91 (0.51–1.61)
Easiest option	0.82 (0.55–1.21)	0.55 (0.34–0.88)	1	0.77 (0.50–1.19)	2.10 (1.29–3.39)
Need arose outside office hours	1.04 (0.99–1.09)	1.04 (0.99–1.10)	1	1.03 (0.98–1.09)	0.96 (0.89–1.04)
Need for quick help because of work	0.25 (0.17–0.36)	0.17 (0.10–0.26)	1	0.66 (0.46–0.93)	0.15 (0.07–0.30)
Previous experience and knowledge					
Previous experience with similar symptoms	1.12 (0.81–1.54)	1.60 (1.14–2.26)	1	1.49 (1.06–2.10)	2.56 (1.68–3.90)
Previous positive experience with this healthcare service	1.41 (1.16–1.72)	1.20 (0.96–1.50)	1	1.11 (0.89–1.39)	1.18 (0.90–1.54)
Perceived most suitable healthcare service	1.38 (1.00–1.89)	1.38 (0.97–1.95)	1	1.91 (1.33–2.73)	2.77 (1.67–4.60)
Needed second opinion	1.17 (0.63–2.18)	0.66 (0.30–1.44)	1	0.75 (0.35–1.58)	1.48 (0.65–3.39)
Needs and wishes					
Did not know what to do	0.70 (0.52–0.93)	0.50 (0.36–0.69)	1	0.59 (0.43–0.80)	0.84 (0.56–1.28)
Did not know where else to call	0.70 (0.52–0.95)	0.47 (0.33–0.67)	1	0.64 (0.46–0.89)	1.28 (0.84–1.94)
Wanted to talk to a physician	1.21 (0.89–1.67)	1.13 (0.80–1.61)	1	1.44 (1.01–2.05)	2.83 (1.66–4.81)
Wanted to talk to a nurse	1.19 (0.91–1.56)	1.06 (0.79–1.56)	1	1.07 (0.79–1.44)	1.00 (0.67–1.50)
Could not take responsibility	0.78 (0.63–0.97)	0.89 (0.71–1.13)	1	1.22 (1.00–1.49)	1.71 (1.38–2.11)
Nobody to talk to	1.00 (0.53–1.88)	1.32 (0.70–2.45)	1	1.04 (0.55–1.98)	2.47 (1.35–4.52)
Recommended (from non-medical person) to call	0.30 (0.23–0.40)	0.25 (0.18–0.36)	1	0.48 (0.37–0.62)	0.84 (0.65–1.08)

*Adjusted for ethnicity, education level, marital status, and self-perceived health.

More often than other age groups, young adults ‘did not know what to do’ (PRR: 0–4 years: 0.70 (CI: 0.52–0.93), 5–12 years: 0.50 (CI: 0.36–0.69), 40–64 years: 0.59 (CI: 0.43–0.80), >64 years: 0.84 (CI: 0.56–1.28)) or ‘did not know who else to call’ (PRR: 0–4 years: 0.70 (CI: 0.52–0.95), 5–12 years: 0.47 (CI: 0.33–0.67), 40–64 years: 0.64 (CI: 0.46–0.89)). Additionally, the group of young adults less often reported ‘previous experiences with similar symptoms’ as a motive for calling (PRR: 0–4 years: 1.12 (CI: 0.81–1.54), 5–12 years: 1.60 (CI: 1.14–2.26), 40–64 years: 1.49 (CI: 1.06–2.10), >64 years: 2.56 (CI: 1.68–3.90)).

## Discussion

### Principal findings

This study explored the motives for contacting OOH-PC across age groups, finding associations between certain motives and specific age groups. Motives related to perceived barriers and benefits, such as ‘Own GP no time available soon enough’ or ‘Need for quick help because of work’ were statistically significantly more important for young adults compared with other age groups.

Motives related to own assessment and expectations were more important for older patients, such as ‘perceived condition to be life-threatening’. ‘Worried’ was significantly more important as a motive for calling among parents of young children and patients aged >64 years compared with other age groups. Concerning needs and wishes, young adults more often than other age groups stated ‘recommended to call’ and ‘did not know what to do’ as a motive for calling, whereas ‘previous experience with similar symptom’ less often formed a motive in this age group.

### Strengths and limitations of the study

The large number of included respondents in our study exploring patient motives in two regions in Denmark made it feasible to obtain precise estimates. The systematic development of the questionnaire was an additional strength; the development was based on relevant literature on decision-making motives and inspired by a thoroughly worked out model [17]. Moreover, the questionnaire proved to have good face and content validity; this was evaluated through discussions with experts and pilot tests among patients. Presenting 26 predefined motives for contacting OOH-PC gave the patients good possibility to select multiple relevant motives and to assess their importance.

Although relatively low, the response rates were acceptable for this type of study (47.1%). A risk of selection bias cannot be excluded as we found that parents of young children and adults aged 18–39 years significantly more often did not respond, while patients aged >64 years responded more often. Such age-related variations in response rates have also been found in previous studies [19], especially the low rate in young adults below 40 years of age [20]. This pattern suggests that many young adults do not give priority to responding, perhaps due to lack of time. Thus, our finding that young adults and parents of young children more often contact OOH-PC due to work-related issues or problems with accessing the GP may reflect an underrepresentation.

The response rate for patients aged 18–39 years and 40–64 years were statistically significantly lower in the Capital Region of Denmark compared with the Central Denmark Region, and this could be a potential source of selection bias. As young adults more often than other age groups stated motives such as ‘Own GP no time available soon enough’ or ‘Need for quick help because of work’, the underrepresentation of young responding adults in the Capital Region of Denmark may constitute an underestimated contribution.

Information bias and recall bias may have been introduced as we collected the data after the contact. This means that the respondents’ answers may have been influenced by their experience of the provided care rather than their actual motives for contacting. However, as the respondents’ actual experience could influence their answers in different ways, by confirming their decision to call as well as weaken the need for calling, it is challenging to take this potential risk of bias into consideration. Moreover, the choice to dichotomize answers and categorize patients into age groups may have concealed fine nuances of responses. However, this categorization was necessary to make the analyses possible, and it added to the clarity of the results.

### Findings in relation to other studies

Motives such as worry and need for reassurance, which were found in our study, have previously been shown to cause medical help-seeking in citizens [13,21,22]. Such motives are present when the OOH-PC services are contacted due to the patient’s fear of losing control of the situation or to rule out serious disease [23,24]. The perspective of the patients, not least parents of sick children, may be very different from that of the healthcare professionals assessing the medical relevance and severity of the problem [25].

Our finding that young adults more often than other age groups stated motives related to perceived barriers and benefits (e.g. accessibility of own GP, restrictions due to work and daycare) may indicate that the OOH-PC services are used for convenience and not only for urgent health problems [25–27], reflecting a modern ‘24/7 access’ expectation, i.e. the OOH-PC services can be used whenever needed instead of contacting the daytime GP [22]. Yet, lack of availability and accessibility of the own GP has shown to be associated with higher use of OOH-PC [15]. The significant difference found between young adults and parents of young children is interesting, as these respondents are largely of the same age, although the parents may be a more homogenous group compared with young adults aged 18–39 years.

In addition, young adults also stated more motives on needs and wishes (e.g. recommendation, did not know what to do), which may indicate a knowledge gap as well as societal changes. Knowledge on acute health problems is partly collected through a social network. Also information on how to navigate the healthcare system may be introduced through family networks, which has been changed with both smaller families and more distance to family members.

Our finding that older patients more often than younger adults stated having nobody to talk to and not wanting to take responsibility is in line with their socio-economic status. Many older people live alone, while they at the same time have a higher risk of getting an acute severe condition such as stroke and myocardial infarction [28]. Due to ageing and improved healthcare, many older patients suffer from co-morbidity [29,30]. This seems to relate to having more often motives related to own assessment and expectations (e.g. life-threatening condition and need of care).

### Implications for health policy and future research

It is unclear whether the finding of young adults stating motives related to barriers to get help when perceived needed more often than other age groups is related to different expectations of availability of healthcare (24/7) or to accessibility issues. Future studies should explore the extent of this attitude in patients and its potential association with age, including health policy perspectives as to whether OOH-PC services are still intended to exclusively target urgent health problems.

### Conclusion

The present study adds to the knowledge on patients’ motives for contacting OOH-PC services and discloses that a number of different motives influence the patient’s decision. Remarkable differences between age groups were seen, singling out young adults as different from the other age groups, as young adults more often stated motives related to perceived barriers, such as ‘Own GP no time available soon enough’ and ‘Need for quick help because of work’ compared with most other age groups. This indicates a change in expectations to availability of and accessibility to healthcare in future generations.

## Supplementary Material

Supplemental Material
